# *N*′-[1-(2-Amino­phen­yl)ethyl­idene]benzo­hydrazide

**DOI:** 10.1107/S1600536810010937

**Published:** 2010-04-24

**Authors:** Vinod P. Singh, Shweta Singh

**Affiliations:** a Faculty of Science, Department of Chemistry, Banaras Hindu University, Varanasi, U. P. 221 005, India

## Abstract

The title compound, C_15_H_15_N_3_O, was obtained by a condensation reaction between *o*-amino­acetophenone and benzoyl hydrazine. The mol­ecule displays an *E* configuration about the C=N bond. Intra­molecular N—H⋯N hydrogen bonds are formed between the 2-amino­phenyl and imine groups. In the crystal, dimers are formed between mol­ecules linked by inter­molecular N—H⋯O hydrogen bonds from the 2-amino­phenyl group. In addition there are inter­molecular N—H⋯O hydrogen bonds between the amine and carbonyl groups of adjacent mol­ecules. The mol­ecule is twisted rather than planar due to a steric inter­action between the central amide group and the two outer benzene rings. The dihedral angles between this central group and the two rings are 23.29 (9) and 24.96 (9)°.

## Related literature

For the biological properties of hydrazones derived from the condensation reactions of hydrazides with aldehydes or ketones, see: Gupta *et al.* (2007 ▶); Kocyigit-Kaymakcioglu *et al.* (2009 ▶); Kou *et al.* (2009 ▶); Mahalingam *et al.* (2009 ▶); Sundaraval *et al.* (2009 ▶); Yin *et al.* (2007 ▶); Zhang *et al.* (2007 ▶). For related structures, see: Fun *et al.* (2008*a* ▶,*b* ▶); Qiu & Zhao (2008 ▶); Qiu (2009 ▶); Ren (2009 ▶); Xiao & Wei (2009 ▶).
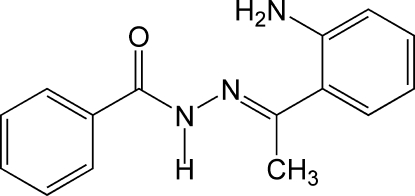


## Experimental

### 

#### Crystal data

C_15_H_15_N_3_O
*M* *_r_* = 253.30
Monoclinic, 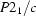

*a* = 13.7531 (10) Å
*b* = 5.1575 (3) Å
*c* = 18.7178 (13) Å
β = 105.917 (7)°
*V* = 1276.78 (15) Å^3^
*Z* = 4
Mo *K*α radiation
μ = 0.09 mm^−1^
*T* = 293 K
0.33 × 0.25 × 0.13 mm

#### Data collection

Oxford Diffraction Xcalibur Eos diffractometer
Absorption correction: multi-scan (*CrysAlis PRO*; Oxford Diffraction, 2009 ▶) *T*_min_ = 0.780, *T*_max_ = 1.000
5257 measured reflections
2894 independent reflections
1839 reflections with *I* > 2σ(*I*)
*R*_int_ = 0.016

#### Refinement

*R*[*F* ^2^ > 2σ(*F* ^2^)] = 0.042
*wR*(*F* ^2^) = 0.125
*S* = 0.98
2894 reflections
173 parameters
H-atom parameters constrained
Δρ_max_ = 0.23 e Å^−3^
Δρ_min_ = −0.17 e Å^−3^

### 

Data collection: *CrysAlis PRO* (Oxford Diffraction, 2009 ▶); cell refinement: *CrysAlis PRO*; data reduction: *CrysAlis PRO*; program(s) used to solve structure: *SHELXS97* (Sheldrick, 2008 ▶); program(s) used to refine structure: *SHELXL97* (Sheldrick, 2008 ▶); molecular graphics: *ORTEP-3* (Farrugia, 1999 ▶); software used to prepare material for publication: *CIFTAB* (Sheldrick, 2008 ▶).

## Supplementary Material

Crystal structure: contains datablocks I, global. DOI: 10.1107/S1600536810010937/bv2135sup1.cif

Structure factors: contains datablocks I. DOI: 10.1107/S1600536810010937/bv2135Isup2.hkl

Additional supplementary materials:  crystallographic information; 3D view; checkCIF report

## Figures and Tables

**Table 1:** Hydrogen-bond geometry (Å, °)

*D*—H⋯*A*	*D*—H	H⋯*A*	*D*⋯*A*	*D*—H⋯*A*
N3—H3*B*⋯O1^i^	0.86	2.41	3.1611 (15)	147
N1—H1*A*⋯O1^ii^	0.86	2.29	3.0856 (16)	154
N1—H1*B*⋯N2	0.86	2.03	2.6626 (16)	130

Symmetry codes: (i) 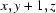; (ii) 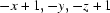.

**Fig. 1.**
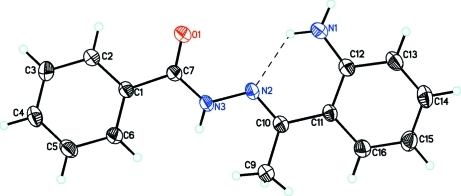
Molecular diagram with labeled atoms of Benzoic acid [1-(2-amino-phenyl)- ethylidene]-hydrazide. Hydrogen bonds are shown by dashed lines.

**Fig. 2.**
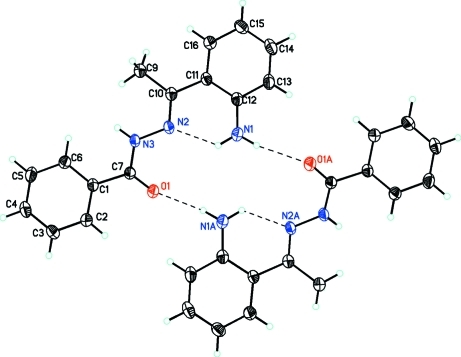
Diagram showing the formation of in-plane intermolecular hydrogen-bonded dimers. Hydrogen bonds are shown by dashed lines.

**Fig. 3.**
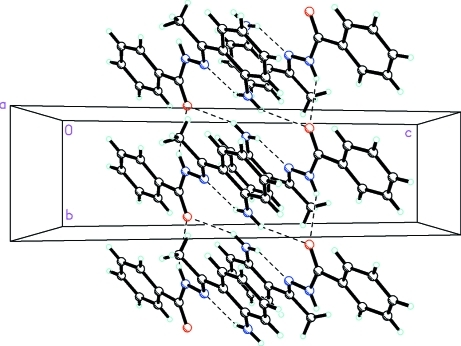
Packing diagram showing both in-plane and out of plane intermolecular hydrogen bonding. Hydrogen bonds are shown by dashed lines.

## supplementary crystallographic information

### Comment

Hydrazones derived from the condensation reactions of hydrazides with aldehydes or ketones show excellent biological properties, such as antimicrobial, antitubercular, anticancer and antimalarial (Kocyigit-Kaymakcioglu *et al.*, 2009; Kou *et al.*, 2009; Mahalingam *et al.*., 2009; Sundaravel *et al.*, 2009; Yin *et al.*, 2007; Zhang *et al.*,2007. The hydrazones are also important for their use as plasticizers and stabilizers for polymers, polymerization initiators, antioxidants and as indicators (Gupta *et al.*, 2007). Recently, a large number of hydrazone compounds have been reported (Qiu *et al.*, 2008; Qiu, 2009; Ren *et al.*, 2009). In this paper, a new hydrazone compound, derived from the condensation reaction of 2-aminoacetophenone and benzoyl hydrazine, has been reported.

The molecular structure of the title compound is shown in the fig. 1. The molecule and displays an E configuration about the C=N double bond. All bond lengths are within normal ranges (Xiao *et al.*, 2009; Fun *et al.*, 2008a,b). The molecular conformation is stabilized by an intramolecular N—H···.O hydrogen bond and short contact bonds (Fig. 1). In the crystal there are both inter- and intra-molecular hydrogen bonding involving the amine protons. In-plane dimers (r.m.s. deviation for N1 N2 N3 C10–C16 and equivalent atoms = 0.016 Å) are formed between molecules linked by N—H···O hydrogen bonds from the 2-aminophenyl moiety (Fig. 2). In addition there are intermolecular out of plane N—H···O hydrogen bonds between amine and carbonyl group of adjoining molecules (Fig. 3). Intramolecular N—H···N hydrogen bonds are formed between the 2-aminophenyl and imine moieties within the same molecule. The molecule is twisted rather than planar due to steric interaction between the central amide group and the two end groups. The torsion angles between this central group and the two ends are 23.29 (9) and 24.96 (9)° respectively.

### Experimental

An ethanolic solution of benzoyl hydazine (50 ml, 6.8 g) was taken in a round bottom flask followed by dropwise addition of ethanolic solution of *o*-aminoacetophenone (50 ml, 6.05 ml) with stirring. The above solution was refluxed for 4-5 h and gave a yellow transparent solution. On keeping the solution in open air for 5-6 h in a beaker, yellow crystals of the product were obtained.

### Refinement

H atoms bound to C and N atoms were located in a difference Fourier map, refined isotropically and then placed using HFIX commands in SHELXL97. All H atoms were allowed for as riding atoms with the N—H distances of 0.86 Å, and C—H distances of 0.93 and 0.96 (2) Å with *U*ĩso~(H) = 1.2 [1.5*U*_eq_(C) for CH_3_].

### Crystal data

**Table:**

C_15_H_15_N_3_O	*F*(000) = 536
*M**_r_* = 253.30	*D*_x_ = 1.318 Mg m^−^^3^
Monoclinic, *P*2_1_/*c*	Melting point: 449 K
Hall symbol: -P 2ybc	Mo *K*α radiation, λ = 0.71073 Å
*a* = 13.7531 (10) Å	Cell parameters from 2381 reflections
*b* = 5.1575 (3) Å	θ = 2.1–28.9°
*c* = 18.7178 (13) Å	µ = 0.09 mm^−^^1^
β = 105.917 (7)°	*T* = 293 K
*V* = 1276.78 (15) Å^3^	Block, yellow
*Z* = 4	0.33 × 0.25 × 0.13 mm

### Data collection

**Table:**

Oxford Diffraction Xcalibur Eos diffractometer	2894 independent reflections
Radiation source: fine-focus sealed tube	1839 reflections with *I* > 2σ(*I*)
graphite	*R*_int_ = 0.016
Detector resolution: 16.0938 pixels mm^-1^	θ_max_ = 28.9°, θ_min_ = 2.3°
ω scans	*h* = −18→18
Absorption correction: multi-scan (*CrysAlis PRO*; Oxford Diffraction, 2009)	*k* = 0→6
*T*_min_ = 0.780, *T*_max_ = 1.000	*l* = 0→25
5257 measured reflections	

### Refinement

**Table:**

Refinement on *F*^2^	Primary atom site location: structure-invariant direct methods
Least-squares matrix: full	Secondary atom site location: difference Fourier map
*R*[*F*^2^ > 2σ(*F*^2^)] = 0.042	Hydrogen site location: inferred from neighbouring sites
*wR*(*F*^2^) = 0.125	H-atom parameters constrained
*S* = 0.98	*w* = 1/[σ^2^(*F*_o_^2^) + (0.0722*P*)^2^] where *P* = (*F*_o_^2^ + 2*F*_c_^2^)/3
2894 reflections	(Δ/σ)_max_ < 0.001
173 parameters	Δρ_max_ = 0.23 e Å^−^^3^
0 restraints	Δρ_min_ = −0.17 e Å^−^^3^

### Special details

**Table:**

Geometry. All esds (except the esd in the dihedral angle between two l.s. planes) are estimated using the full covariance matrix. The cell esds are taken into account individually in the estimation of esds in distances, angles and torsion angles; correlations between esds in cell parameters are only used when they are defined by crystal symmetry. An approximate (isotropic) treatment of cell esds is used for estimating esds involving l.s. planes.
Refinement. Refinement of *F*^2^ against ALL reflections. The weighted *R*-factor wR and goodness of fit *S* are based on *F*^2^, conventional *R*-factors *R* are based on *F*, with *F* set to zero for negative *F*^2^. The threshold expression of *F*^2^ > σ(*F*^2^) is used only for calculating *R*-factors(gt) etc. and is not relevant to the choice of reflections for refinement. *R*-factors based on *F*^2^ are statistically about twice as large as those based on *F*, and *R*- factors based on ALL data will be even larger.

### Fractional atomic coordinates and isotropic or equivalent isotropic displacement parameters (Å^2^)

**Table:**

	*x*	*y*	*z*	*U*_iso_*/*U*_eq_	
O1	0.66011 (8)	0.1102 (2)	0.65390 (6)	0.0519 (3)	
N1	0.39154 (10)	0.1668 (2)	0.49800 (7)	0.0503 (4)	
H1A	0.3786	0.0452	0.4653	0.060*	
H1B	0.4518	0.1849	0.5267	0.060*	
N2	0.50765 (9)	0.4528 (2)	0.60680 (7)	0.0421 (3)	
N3	0.60065 (8)	0.5188 (2)	0.65630 (6)	0.0412 (3)	
H3B	0.6115	0.6736	0.6738	0.049*	
C1	0.76802 (10)	0.4196 (2)	0.73150 (8)	0.0358 (3)	
C2	0.85408 (11)	0.2716 (3)	0.73861 (8)	0.0447 (4)	
H2A	0.8526	0.1323	0.7068	0.054*	
C3	0.94215 (11)	0.3296 (3)	0.79275 (9)	0.0525 (4)	
H3A	0.9999	0.2306	0.7966	0.063*	
C4	0.94508 (12)	0.5322 (3)	0.84082 (9)	0.0511 (4)	
H4A	1.0042	0.5680	0.8777	0.061*	
C5	0.86097 (12)	0.6814 (3)	0.83443 (9)	0.0515 (4)	
H5A	0.8632	0.8193	0.8668	0.062*	
C6	0.77205 (11)	0.6280 (3)	0.77967 (8)	0.0440 (4)	
H6A	0.7153	0.7313	0.7752	0.053*	
C7	0.67244 (10)	0.3368 (3)	0.67595 (8)	0.0369 (3)	
C9	0.43928 (12)	0.8049 (3)	0.66631 (9)	0.0550 (4)	
H9A	0.4936	0.7669	0.7097	0.083*	
H9B	0.3770	0.8173	0.6799	0.083*	
H9C	0.4525	0.9664	0.6453	0.083*	
C10	0.43119 (10)	0.5926 (3)	0.61032 (7)	0.0367 (3)	
C11	0.33225 (10)	0.5346 (2)	0.55733 (7)	0.0357 (3)	
C12	0.31699 (11)	0.3301 (3)	0.50418 (8)	0.0391 (3)	
C13	0.21932 (12)	0.2954 (3)	0.45633 (9)	0.0508 (4)	
H13A	0.2085	0.1638	0.4210	0.061*	
C14	0.13992 (13)	0.4485 (3)	0.45999 (10)	0.0571 (5)	
H14A	0.0762	0.4184	0.4279	0.069*	
C15	0.15369 (12)	0.6478 (3)	0.51108 (9)	0.0526 (4)	
H15A	0.0997	0.7528	0.5135	0.063*	
C16	0.24806 (11)	0.6886 (3)	0.55826 (9)	0.0468 (4)	
H16A	0.2568	0.8237	0.5923	0.056*	

### Atomic displacement parameters (Å^2^)

**Table:**

	*U*^11^	*U*^22^	*U*^33^	*U*^12^	*U*^13^	*U*^23^
O1	0.0476 (6)	0.0412 (6)	0.0576 (7)	0.0001 (5)	−0.0013 (5)	−0.0141 (5)
N1	0.0502 (8)	0.0459 (7)	0.0476 (8)	−0.0006 (6)	0.0015 (6)	−0.0132 (6)
N2	0.0344 (6)	0.0422 (7)	0.0416 (7)	−0.0020 (5)	−0.0033 (5)	−0.0061 (5)
N3	0.0364 (7)	0.0354 (6)	0.0431 (7)	−0.0027 (5)	−0.0035 (5)	−0.0068 (5)
C1	0.0367 (7)	0.0334 (7)	0.0342 (7)	−0.0036 (6)	0.0044 (6)	0.0011 (6)
C2	0.0416 (8)	0.0453 (8)	0.0434 (9)	0.0002 (7)	0.0052 (6)	−0.0068 (7)
C3	0.0375 (8)	0.0583 (10)	0.0549 (10)	0.0018 (7)	0.0012 (7)	−0.0017 (8)
C4	0.0436 (9)	0.0545 (10)	0.0455 (9)	−0.0107 (8)	−0.0040 (7)	−0.0004 (8)
C5	0.0579 (10)	0.0447 (9)	0.0448 (9)	−0.0076 (7)	0.0023 (7)	−0.0101 (7)
C6	0.0438 (8)	0.0378 (8)	0.0462 (9)	0.0002 (6)	0.0052 (7)	−0.0044 (7)
C7	0.0365 (7)	0.0360 (8)	0.0361 (8)	−0.0017 (6)	0.0063 (6)	−0.0031 (6)
C9	0.0441 (9)	0.0630 (10)	0.0519 (10)	0.0027 (8)	0.0029 (7)	−0.0175 (8)
C10	0.0389 (8)	0.0352 (7)	0.0337 (7)	−0.0045 (6)	0.0064 (6)	0.0020 (6)
C11	0.0351 (7)	0.0345 (7)	0.0337 (7)	−0.0034 (6)	0.0028 (6)	0.0034 (6)
C12	0.0421 (8)	0.0357 (7)	0.0359 (8)	−0.0038 (6)	0.0043 (6)	0.0043 (6)
C13	0.0519 (9)	0.0489 (9)	0.0424 (9)	−0.0075 (8)	−0.0026 (7)	−0.0052 (7)
C14	0.0427 (9)	0.0637 (10)	0.0527 (10)	−0.0068 (8)	−0.0075 (7)	0.0038 (8)
C15	0.0400 (9)	0.0576 (10)	0.0544 (10)	0.0056 (7)	0.0029 (7)	0.0036 (8)
C16	0.0441 (9)	0.0453 (9)	0.0468 (9)	0.0016 (7)	0.0051 (7)	−0.0028 (7)

### Geometric parameters (Å, °)

**Table:**

O1—C7	1.2359 (16)	C5—H5A	0.9300
N1—C12	1.3561 (17)	C6—H6A	0.9300
N1—H1A	0.8600	C9—C10	1.498 (2)
N1—H1B	0.8600	C9—H9A	0.9600
N2—C10	1.2915 (18)	C9—H9B	0.9600
N2—N3	1.4002 (14)	C9—H9C	0.9600
N3—C7	1.3387 (17)	C10—C11	1.4781 (18)
N3—H3B	0.8600	C11—C16	1.4079 (19)
C1—C2	1.3838 (19)	C11—C12	1.4254 (19)
C1—C6	1.3942 (19)	C12—C13	1.4068 (19)
C1—C7	1.4972 (18)	C13—C14	1.364 (2)
C2—C3	1.382 (2)	C13—H13A	0.9300
C2—H2A	0.9300	C14—C15	1.381 (2)
C3—C4	1.372 (2)	C14—H14A	0.9300
C3—H3A	0.9300	C15—C16	1.371 (2)
C4—C5	1.367 (2)	C15—H15A	0.9300
C4—H4A	0.9300	C16—H16A	0.9300
C5—C6	1.391 (2)		
			
C12—N1—H1A	120.0	C10—C9—H9A	109.5
C12—N1—H1B	120.0	C10—C9—H9B	109.5
H1A—N1—H1B	120.0	H9A—C9—H9B	109.5
C10—N2—N3	116.06 (11)	C10—C9—H9C	109.5
C7—N3—N2	118.84 (11)	H9A—C9—H9C	109.5
C7—N3—H3B	120.6	H9B—C9—H9C	109.5
N2—N3—H3B	120.6	N2—C10—C11	117.74 (12)
C2—C1—C6	118.80 (13)	N2—C10—C9	122.56 (13)
C2—C1—C7	118.32 (12)	C11—C10—C9	119.69 (13)
C6—C1—C7	122.72 (13)	C16—C11—C12	117.51 (12)
C3—C2—C1	120.37 (14)	C16—C11—C10	119.11 (13)
C3—C2—H2A	119.8	C12—C11—C10	123.39 (13)
C1—C2—H2A	119.8	N1—C12—C13	118.61 (13)
C4—C3—C2	120.52 (15)	N1—C12—C11	123.31 (12)
C4—C3—H3A	119.7	C13—C12—C11	118.07 (13)
C2—C3—H3A	119.7	C14—C13—C12	122.14 (15)
C5—C4—C3	119.95 (14)	C14—C13—H13A	118.9
C5—C4—H4A	120.0	C12—C13—H13A	118.9
C3—C4—H4A	120.0	C13—C14—C15	120.38 (15)
C4—C5—C6	120.33 (15)	C13—C14—H14A	119.8
C4—C5—H5A	119.8	C15—C14—H14A	119.8
C6—C5—H5A	119.8	C16—C15—C14	119.08 (15)
C5—C6—C1	120.02 (14)	C16—C15—H15A	120.5
C5—C6—H6A	120.0	C14—C15—H15A	120.5
C1—C6—H6A	120.0	C15—C16—C11	122.81 (14)
O1—C7—N3	123.24 (12)	C15—C16—H16A	118.6
O1—C7—C1	121.04 (12)	C11—C16—H16A	118.6
N3—C7—C1	115.59 (12)		
			
C10—N2—N3—C7	155.32 (13)	N3—N2—C10—C9	−3.0 (2)
C6—C1—C2—C3	0.2 (2)	N2—C10—C11—C16	−178.10 (13)
C7—C1—C2—C3	−175.37 (14)	C9—C10—C11—C16	2.9 (2)
C1—C2—C3—C4	0.9 (2)	N2—C10—C11—C12	1.4 (2)
C2—C3—C4—C5	−1.3 (3)	C9—C10—C11—C12	−177.57 (13)
C3—C4—C5—C6	0.4 (3)	C16—C11—C12—N1	−179.41 (13)
C4—C5—C6—C1	0.7 (2)	C10—C11—C12—N1	1.1 (2)
C2—C1—C6—C5	−1.0 (2)	C16—C11—C12—C13	0.0 (2)
C7—C1—C6—C5	174.34 (14)	C10—C11—C12—C13	−179.53 (13)
N2—N3—C7—O1	−1.7 (2)	N1—C12—C13—C14	178.76 (15)
N2—N3—C7—C1	−177.50 (12)	C11—C12—C13—C14	−0.7 (2)
C2—C1—C7—O1	22.1 (2)	C12—C13—C14—C15	0.9 (3)
C6—C1—C7—O1	−153.29 (15)	C13—C14—C15—C16	−0.3 (3)
C2—C1—C7—N3	−162.04 (13)	C14—C15—C16—C11	−0.4 (2)
C6—C1—C7—N3	22.6 (2)	C12—C11—C16—C15	0.5 (2)
N3—N2—C10—C11	178.06 (11)	C10—C11—C16—C15	−179.94 (14)

### Hydrogen-bond geometry (Å, °)

**Table:**

*D*—H···*A*	*D*—H	H···*A*	*D*···*A*	*D*—H···*A*
N3—H3B···O1^i^	0.86	2.41	3.1611 (15)	147
N1—H1A···O1^ii^	0.86	2.29	3.0856 (16)	154
N1—H1B···N2	0.86	2.03	2.6626 (16)	130

Symmetry codes: (i) *x*, *y*+1, *z*; (ii) −*x*+1, −*y*, −*z*+1.
